# Modulation of Antioxidant Defense System Is Associated with Combined Drought and Heat Stress Tolerance in Citrus

**DOI:** 10.3389/fpls.2017.00953

**Published:** 2017-06-07

**Authors:** Sara I. Zandalinas, Damián Balfagón, Vicent Arbona, Aurelio Gómez-Cadenas

**Affiliations:** Departament de Ciències Agràries i del Medi Natural, Universitat Jaume ICastelló de la Plana, Spain

**Keywords:** Carrizo citrange, Cleopatra mandarin, drought, heat, oxidative stress

## Abstract

Drought and high temperatures are two major abiotic stress factors that often occur simultaneously in nature, affecting negatively crop performance and yield. Moreover, these environmental challenges induce oxidative stress in plants through the production of reactive oxygen species (ROS). Carrizo citrange and Cleopatra mandarin are two citrus genotypes with contrasting ability to cope with the combination of drought and heat stress. In this work, a direct relationship between an increased antioxidant activity and stress tolerance is reported. According to our results, the ability of Carrizo plants to efficiently coordinate superoxide dismutase (SOD), ascorbate peroxidase (APX), catalase (CAT), and glutathione reductase (GR) activities involved in ROS detoxification along with the maintenance of a favorable GSH/GSSG ratio could be related to their relative tolerance to this stress combination. On the other hand, the increment of SOD activity and the inefficient GR activation along with the lack of CAT and APX activities in Cleopatra plants in response to the combination of drought and heat stress, could contribute to an increased oxidative stress and the higher sensibility of this citrus genotype to this stress combination.

## Introduction

Environmental stresses cause large economic losses in agriculture every year, constraining crop yield and production. Owing to the consequences of the climate change, different combinations of abiotic stress conditions are severely impacting on plants in the natural field (Mittler, 2006; Suzuki et al., 2014; Zandalinas et al., 2017a). Although research on plants is traditionally based on the study of the responses to single abiotic factors, further effort has been made over the last years to analyze plant responses to different combined stresses, either abiotic or biotic (Suzuki et al., 2014; Zandalinas et al., 2017a). Particularly, drought and heat are considered one of the most frequent abiotic stress combinations that drastically affect global agricultural systems [International Panel of Climate Change (IPCC, 2014)].

Reactive oxygen species are normally produced as a result of aerobic metabolism. However, metabolic imbalances produced by changes in environmental conditions promote the over-accumulation of ROS (Suzuki et al., 2012). In general, abiotic stresses that limit CO_2_ availability due to stomatal closure enhance the accumulation of ROS. Interestingly, while ROS, such as H_2_O_2_, are considered important signal transduction molecules (Baxter et al., 2014; Mittler, 2016), they are also toxic, causing extensive cellular damage and inhibition of photosynthesis (Choudhury et al., 2016). To prevent damage, ROS accumulation is mitigated by the antioxidant machinery including ROS-scavenging enzymes and increased levels of antioxidants such as AsA and GSH, components of the so-called Halliwell-Asada cycle (Mittler et al., 2004). One of the key enzymes of the antioxidant defense system is the SOD, which constitutes the first level of defense against superoxide radicals. SOD-catalyzed $O_{2}^{•-}$ dismutation renders H_2_O_2_ as a reaction product, which in turn is removed by APX and CAT activities (Mittler et al., 2004). APX reduces H_2_O_2_ using AsA as the electron donor and the balance between GSH and GSSG is critical for maintaining a favorable redox status for the detoxification of H_2_O_2_. In addition, GR, the rate-limiting enzyme of AsA–GSH cycle, keeps the GSH/GSSG ratio favorable for AsA reduction (Foyer and Noctor, 2005).

Several studies have reported that the ability of plants to balance ROS production and scavenging is associated to a higher tolerance to different environmental stresses (Hernandez et al., 2000; Lin et al., 2004; Arbona et al., 2008; Martinez et al., 2016). The accumulation of high amount of ROS-response transcripts in plants subjected to different combinations of stress factors, reflects the relevance of ROS as an important component of acclimation pathways during combined stresses (reviewed in Suzuki et al., 2014). For example, it has been suggested the key role of cytosolic APX1 for the acclimation of plants to a combination of drought and heat (Koussevitzky et al., 2008). In that work, *Arabidopsis* mutants deficient in this enzyme (*apx1*), were found to be highly sensitive to this stress combination. Furthermore, ROS–ABA interactions are very important for plant acclimation to stress combination. In this way, previous reports have shown that mutants impaired in the function of the ABA and ROS-regulated protein phosphates 2C (PP2Cs) (*abi-1*) were sensitive to the combined impact of drought and heat, as well as salinity and heat (Suzuki et al., 2016; Zandalinas et al., 2016a). Furthermore, several studies have reported that the expression of different ROS-scavenging enzymes and the accumulation of different antioxidants exhibit a unique mechanism of response during stress combination that is different than that found in response to each individual stress (Rizhsky et al., 2002, 2004; Srivastava et al., 2012; Prasch and Sonnewald, 2013; Rivero et al., 2013; Pandey et al., 2015; Jin et al., 2016).

We recently demonstrated the different ability of two citrus genotypes, Carrizo citrange and Cleopatra mandarin, to tolerate drought and heat applied alone or in combination. Therefore, physiological responses in terms of gas exchange parameters and chlorophyll fluorescence, evidenced the higher susceptibility of Cleopatra mandarin to combined drought and heat conditions (Zandalinas et al., 2016b). Moreover, metabolite profiling of leaves of both citrus genotypes in response to combined drought and heat revealed that the accumulation of secondary metabolites with antioxidant function is associated to sensitivity to this stress combination (Zandalinas et al., 2017b). Therefore, the higher sensitivity of Cleopatra plants required a higher accumulation of protective metabolites oriented to mitigate the damaging effects of stress, including flavonols, flavones, and limonoids (Zandalinas et al., 2017b). However, the role of the antioxidant defense involving ROS-scavenging enzymes in the tolerance of citrus plants to combined drought and heat is currently unknown. Previous reports have associated the ability to modulate the antioxidant system with the tolerance of citrus plants to waterlogging (Arbona et al., 2008; Hossain et al., 2009), salinity (Arbona et al., 2003), or WS (Wu et al., 2006). In general, these investigations concluded that coordinated antioxidant activity associated to increased activities of SOD and CAT, along with a modulation of the AsA–GSH cycle, allowed citrus plants to reduce stress-induced oxidative damage.

The aim of the present work was to determine the importance of the modulation of the antioxidant system in citrus tolerance to the combination of drought and high temperatures. To achieve this, oxidative metabolism and related antioxidants were studied in two citrus genotypes (Carrizo citrange and Cleopatra mandarin) with different ability to cope with this combined stresses (Zandalinas et al., 2016b).

## Materials and Methods

### Plant Material and Growth Conditions

Carrizo citrange (*Poncirus trifoliata* L. Raf. × *Citrus sinensis* L. Osb.) and Cleopatra mandarin (*Citrus reshni* Hort. Ex Tan.) plants were purchased from a commercial nursery (Beniplant S.L., Penyíscola, Spain). One-year-old seedlings of both citrus genotypes were grown in plastic pots filled with perlite and watered three times a week with a half-strength Hoagland solution under greenhouse conditions, with natural photoperiod and day and night temperature averaging 25.0 ± 3.0°C and 18.0 ± 3.0°C, respectively. Then, plants were maintained for 2 weeks in growth chambers to acclimate to a 16-h photoperiod at 25°C and relative moisture at approximately 80%. Temperature and relative moisture were recorded regularly with a portable USB datalogger (OM-EL-WIN-USB, Omega, NJ, United States).

### Stress Treatments and Experimental Designs

**FIGURE 1:**
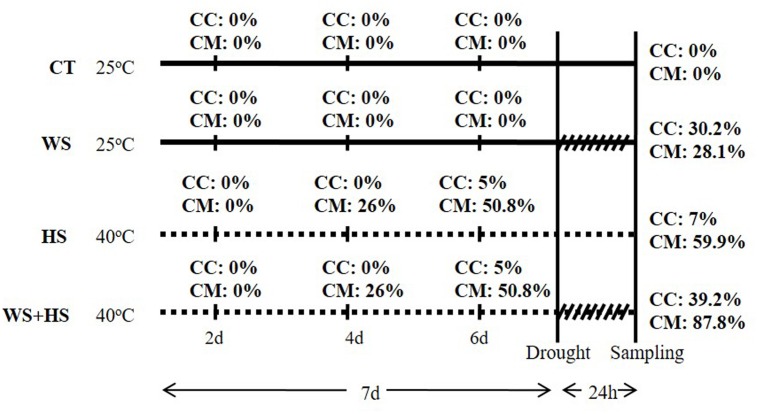
Experimental design used to subject Carrizo and Cleopatra plants to drought (WS), heat stress (HS), and a combination of drought and heat stress (WS+HS) with details of period times for each stress treatment. Percentages of affected leaves in Carrizo (CC) and Cleopatra (CM) subjected to WS, HS, and WS+HS are also indicated.

A 24-h experiment of combined drought and heat was performed with both types of plants (**Figure 1**). High temperatures (40°C) were firstly imposed for 7 days to a group of plants, maintaining another group at 25°C as control. After imposing the temperature treatment, severe WS conditions were applied by transplanting a group of plants grown at 25 or at 40°C to dry perlite. Therefore, four experimental groups for each citrus genotype were established: well-watered plants at 25°C (CT) and at 40°C (HS) and plants subjected to WS at 25°C (WS) and at 40°C (WS+HS). Leaves with an intermediate position in the canopy were harvested and immediately submerged in liquid N_2_.

### Proline Concentration

Proline analysis was performed as described by Bates et al. (1973) with some modifications. Briefly, 50 mg of ground leaf tissue was extracted in 5 ml of 3% sulfosalicylic acid (Panreac, Barcelona, Spain) by sonication for 30 min. After centrifuging at 4000 × *g* for 20 min at 4°C, 1 ml of the supernatant was mixed with 1 ml of glacial acetic acid and ninhydrin reagent (Panreac) in a 1:1 (v:v) ratio. The reaction mixture was incubated in a water bath at 100°C for 1 h and subsequently centrifuged at 2000 × *g* for 5 min at 4°C. Finally, absorbance was read at 520 nm. A standard curve was assayed with pure proline (Sigma-Aldrich, St. Louis, MO, United States).

### Leaf Water Status

Relative water content of citrus leaves was calculated using adjacent leaves, which were weighed to obtain a leaf M_f_. Leaves were allowed to rehydrate overnight in an opaque beaker filled with water. Therefore, they were reweighed to obtain M_t_. Finally, leaves were dried at 80°C for 48 h to obtain M_d_. RWC was calculated as [(M_f_ - M_d_) × (M_t_ - M_d_)^-1^] × 100 according to Morgan (1984).

### Malondialdehyde Concentration

Malondialdehyde content was measured following the procedure of Hodges et al. (1999) with modifications. Ground leaf tissue (0.2 g) were extracted in 2 mL 80% ethanol by sonication for 30 min and, after that, centrifuged at 12000 × *g* for 10 min. Different aliquots of the supernatant were mixed either with 20% trichloroacetic acid or with a mixture of 20% trichloroacetic acid and 0.5% thiobarbituric acid. Both mixtures were incubated in a water bath at 90°C for 1 h. After cooling samples in ice, homogenates were centrifuged at 2000 × *g* for 10 min at 4°C. Lastly, the absorbance at 440, 534, and 600 nm of supernatants was read. The MDA concentration in the extracts was calculated as follows:

(1) [(Abs 532_+TBA_) - (Abs 600_+TBA_) - (Abs 532_-TBA_ - Abs 600_-TBA_)] = A.
(2) [(Abs 440_+TBA_ - Abs 600_+TBA_) × 0.0571] = B
(3) MDA equivalents (nmol ml^-1^) = (A - B/157,000) × 10^6^

MDA concentration was expressed as nmol MDA per gram of fresh weight.

### Gene Expression

The specific primers used for the amplification of each gene are included in Supplementary Table S1. qRT-PCR analyses were performed in a StepOne Real-Time PCR system (Applied Biosystems, Foster City, CA, United States). The reaction mixture contained 1 μL of cDNA, 5 μL of SYBRGreen (Applied Biosystems) and 1 μM of each gene-specific primer pair in a final volume of 10 μL. The thermal profile used to analyze the relative gene expression consisted of 10 min at 95°C for pre-incubation, followed by 40 cycles of 10 s at 95°C for denaturation, 10 s at 60°C for annealing and 20 s at 72°C for extension. Amplicon specificity of the PCR reaction was evaluated by the presence of a single peak in the dissociation curve after the amplification steps. The expression levels of all genes was normalized against the expression of two endogenous control genes (tubulin and actin) based on previous housekeeping selection for citrus tissues (Mafra et al., 2012) and the relative expression were calculated by using REST (Pfaﬄ et al., 2002). For all genes studied, the reference sample was the expression value obtained at the non-stressed samples and set at zero.

### Antioxidant Enzyme Activities

About 100 mg of frozen ground leaf tissue were extracted in 2 mL of phosphate buffer in a ball mill (MillMix20, Domel, Železniki, Slovenija). After centrifugation 14000 × *g* at 4°C for 10 min, supernatant was recovered. Different buffers were used for enzyme extractions as follows: for APX, 50 mM phosphate buffer (pH 7.8) supplemented with 1 mM sodium ascorbate and 1 mM EDTA; for SOD, 50 mM phosphate buffer (pH 6.8) with 1.33 mM diethyl-diamino-pentaacetic acid; finally, CAT and GR were extracted in 50 mM phosphate buffer (pH 6.8 and pH 7.5, respectively). The APX activity was assayed following the depletion in absorbance at 290 nm due to AsA consumption. The SOD activity was determined following the $O_{2}^{•-}$-induced reduction of nitroblue tetrazolium using the xanthine–xanthine oxidase system. CAT was determined using the hydrogen peroxide-dependent reduction of titanium chloride. The GR activity was studied following the increase in absorbance at 412 nm during 2 min as result of the production of the adduct DTNB-GSH after GSSG reduction. The reaction was initiated by adding a suitable aliquot of enzyme extract and the increment in absorbance was recorded during 3 min at 265 nm. Soluble protein content was determined according to Bradford (1976) using BSA as a standard. Enzyme activity was expressed as U mg^-1^ protein. Further details on enzyme assays are provided in Hossain et al. (2009).

### Ascorbate and Glutathione Levels

Procedures for AsA and GSH determinations are described in Hossain et al. (2009). In short, AsA assay is derived from the reduction of Fe^3+^ to Fe^2+^ in acidic solution by AsA. Fe^2+^ forms a red chelate with bipyridyl that absorbs at 525 nm. DHA was calculated by subtracting AsA from total AsA. The DTNB-GSSG reductase recycling process was used to calculate both total (GSH+GSSG) and GSSG levels.

### Statistical Analyses

Data are means of three independent determinations and were subjected to analysis of variance (ANOVA) using a two-way ANOVA with the interaction genotype × stress followed by Tukey *post hoc* test (*P* < 0.05) when a significant difference was detected.

## Results

### Leaf Damage

As shown in **Figure 1**, 24-h of drought applied individually induced visible leaf damage in both citrus genotypes (30 and 28% of Carrizo and Cleopatra leaves, respectively, were injured). Carrizo plants subjected to continuous HS (40°C) were slightly affected, showing only 5 and 7% of total leaves damaged at 6 days and at the end of the experiment, respectively. On the contrary, after 4 days of heat regime, 26% of Cleopatra leaves were damaged, reaching about 60% at the end of the experiment. Plants subjected to a combination of WS+HS showed the highest percentage of leaf damage in both citrus genotypes. Hence, 39 and 88% of leaves were affected by the combined stresses in Carrizo and Cleopatra, respectively (**Figure 1**).

### Water Status

Leaf RWC of Carrizo and Cleopatra plants subjected to drought, HS and a combination of WS+HS was measured (**Table 1**). WS+HS conditions similarly decreased leaf RWC in Carrizo and Cleopatra: in Carrizo plants subjected to WS and HS, RWC reached 60 and 75% respect to control values, respectively. In Cleopatra plants, RWC decreased to 60 and 69% (with respect to controls) in response to WS and HS, respectively. Interestingly, stress combination had an additive impact on this parameter, showing the greatest decrease (43 and 39% with respect to control values in Carrizo and Cleopatra, respectively; **Table 1**).

**Table 1:** Relative water content (RWC) of Carrizo and Cleopatra leaves subjected to drought (WS), heat (HS), and their combination (WS+HS).

Genotype		RWC (%)
Carrizo		
	CT	92.96 ± 0.75 a
	WS	60.32 ± 3.01 bc
	HS	75.32 ± 4.73 b
	WS+HS	43.38 ± 5.17 de
Cleopatra		
	CT	93.72 ± 3.01 a
	WS	59.66 ± 4.31 cd
	HS	69.01 ± 3.92 bcd
	WS+HS	39.41 ± 6.07 e
G: ^∗∗^ S: ^∗∗∗^ G×S: ns		
S: ^∗∗∗^		

*Data are presented as mean value of three different replicates ± SD. Different letters denote statistical significance at *p* ≤ 0.05. G, genotype; S, stress treatment; G×S, interaction genotype × stress treatment. ^∗^*P* < 0.05; ^∗∗^*P* < 0.01; ^∗∗∗^*P* < 0.001; ns, no statistical differences*.

### Proline Concentration

**FIGURE 2:**
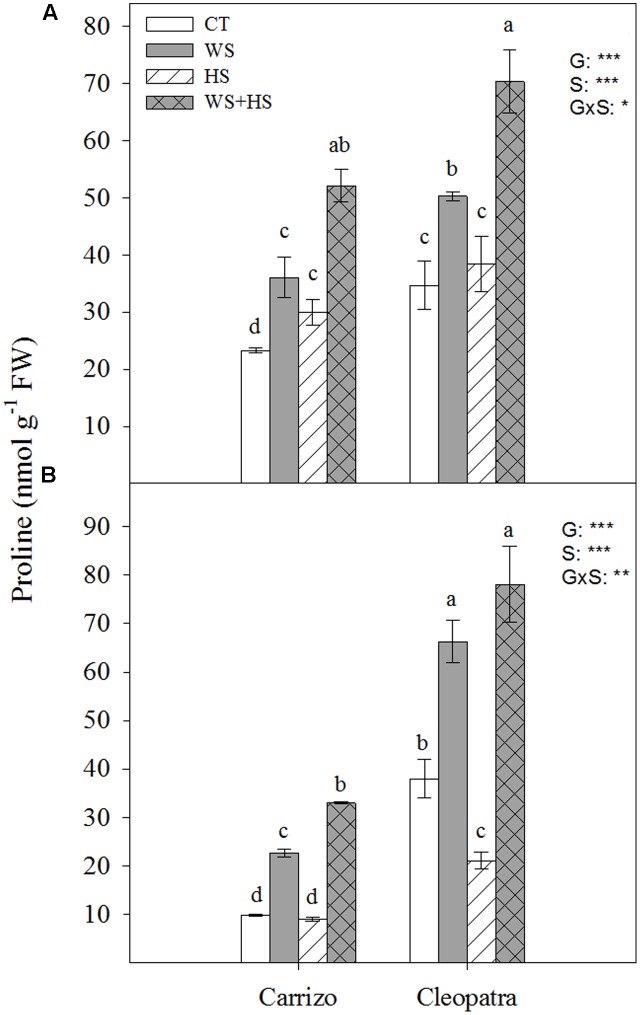
Proline accumulation in leaves **(A)** and roots **(B)** of Carrizo and Cleopatra plants subjected to drought (WS), heat (HS), and a combination of drought and heat stress (WS+HS). Different letters denote statistical significance at *p* ≤ 0.05. G, genotypes; S, stress treatment; G×S, interaction genotype × stress treatment. ^∗^*P* < 0.05; ^∗∗^*P* < 0.01; ^∗∗∗^*P* < 0.001; ns, no statistical differences.

Endogenous proline levels were examined in leaves and roots of both citrus genotypes in response to individual and combined stresses (**Figure 2**). In general, basal proline content of both Cleopatra leaves and roots almost doubled the levels observed in Carrizo. Furthermore, proline concentration in Carrizo leaves significantly increased respect to control values in response to individual stresses. In addition, stress combination induced the highest proline concentration in this genotype. Proline content only increased in response to WS and WS+HS in Cleopatra leaves (**Figure 2A**). On the other hand, significant increments of proline levels were observed in Carrizo roots subjected to WS (2.2-fold) and especially to WS+HS (3.1-fold), whereas HS did not impact on proline build-up. Finally, proline levels increased similarly in Cleopatra roots in response to WS and WS+HS (about two-fold) and HS caused a reduction of its levels below control values (**Figure 2B**).

### MDA Concentration

**FIGURE 3:**
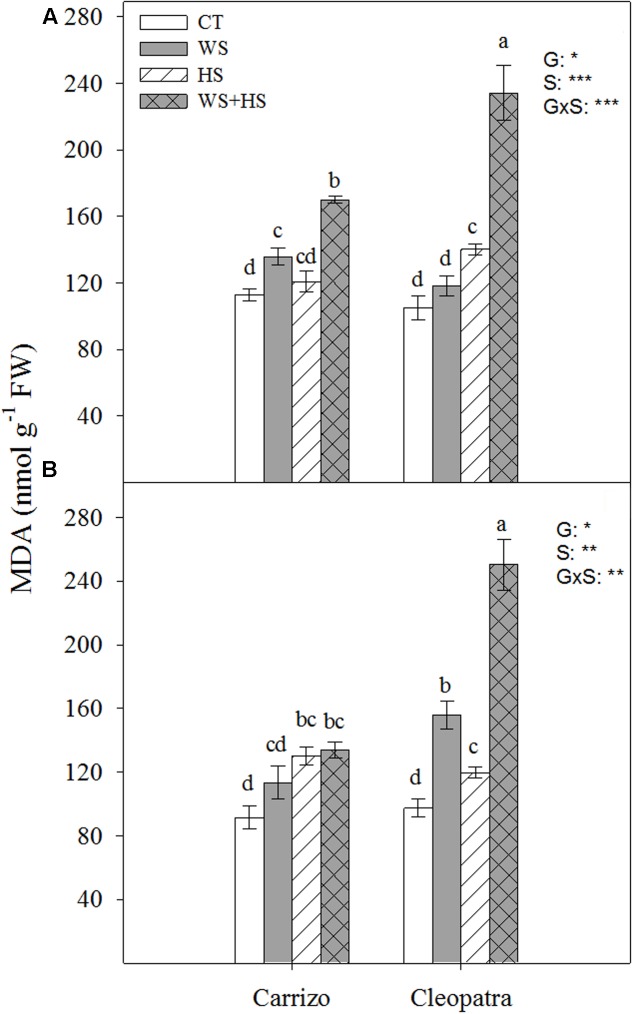
Malondialdehyde (MDA) accumulation in leaves **(A)** and roots **(B)** of Carrizo and Cleopatra plants subjected to drought (WS), heat (HS), and a combination of drought and heat stress (WS+HS). Different letters denote statistical significance at *p* ≤ 0.05. G, genotypes; S, stress treatment; G×S, interaction genotype × stress treatment. ^∗^*P* < 0.05; ^∗∗^*P* < 0.01; ^∗∗∗^*P* < 0.001; ns, no statistical differences.

Oxidative damage in terms of MDA concentration in response to drought, HS and the combination of WS+HS was studied in leaves and roots of both citrus genotypes (**Figure 3**). MDA accumulated in Carrizo leaves in response to WS and more prominently in response to WS+HS. On the contrary, Cleopatra leaves increased MDA content in response to HS and especially during WS+HS (**Figure 3A**). MDA accumulation pattern in roots was different between both citrus genotypes. Whereas WS induced MDA accumulation only in Cleopatra, HS slightly increased its accumulation in both citrus plants. Strikingly, stress combination resulted in a minor MDA accumulation in Carrizo roots whereas in Cleopatra roots, it resulted in a strong MDA accumulation (**Figure 3B**).

### Antioxidant Enzymatic Activity

**FIGURE 4:**
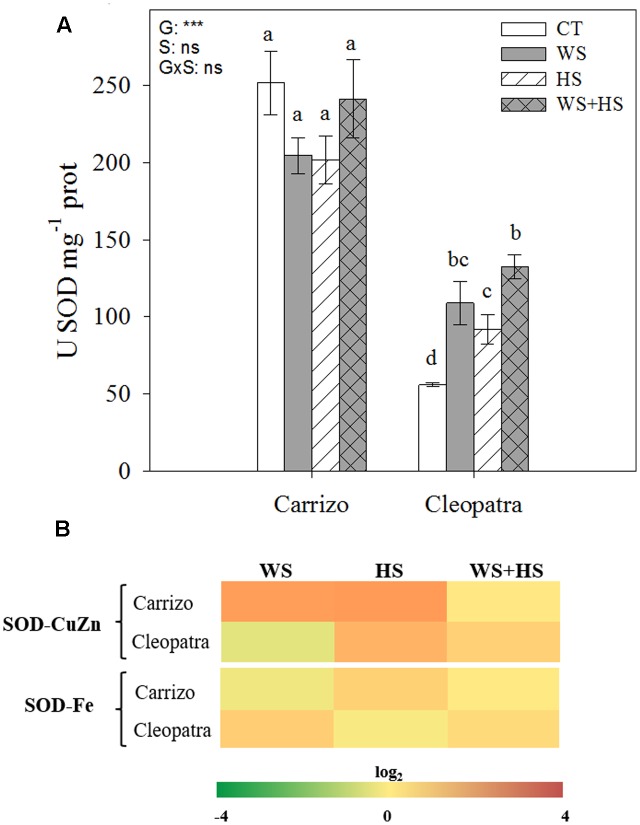
Effects of drought (WS), heat (HS), and a combination of drought and heat stress (WS+HS) on SOD activity **(A)** and transcript expression **(B)** in leaves of Carrizo and Cleopatra plants. Different letters denote statistical significance at *p* ≤ 0.05. G, genotypes; S, stress treatment; G×S, interaction genotype × stress treatment. ^∗^*P* < 0.05; ^∗∗^*P* < 0.01; ^∗∗∗^*P* < 0.001; ns, no statistical differences. Scale for gene expression is log_2_ of the mean values after normalization against control plants.

Under all conditions (control or stress), the SOD activity was significantly higher in Carrizo (five-fold) compared to Cleopatra plants. Imposition of individual and combined stresses had no significant impact on SOD activity in Carrizo leaves, whereas Cleopatra plants showed a two-fold and three-fold increment of this enzymatic activity in response to individual and combined stresses, respectively (**Figure 4A**). Furthermore, the relative expression of the gene encoding SOD-CuZn in Carrizo was up-regulated under individual stress conditions. In Cleopatra leaves, an accumulation of SOD-CuZn transcripts was observed in response to HS and WS+HS treatments. In addition, SOD-Fe transcripts slightly accumulated in response to HS in Carrizo and in response to WS and WS+HS in Cleopatra (**Figure 4B**).

**FIGURE 5:**
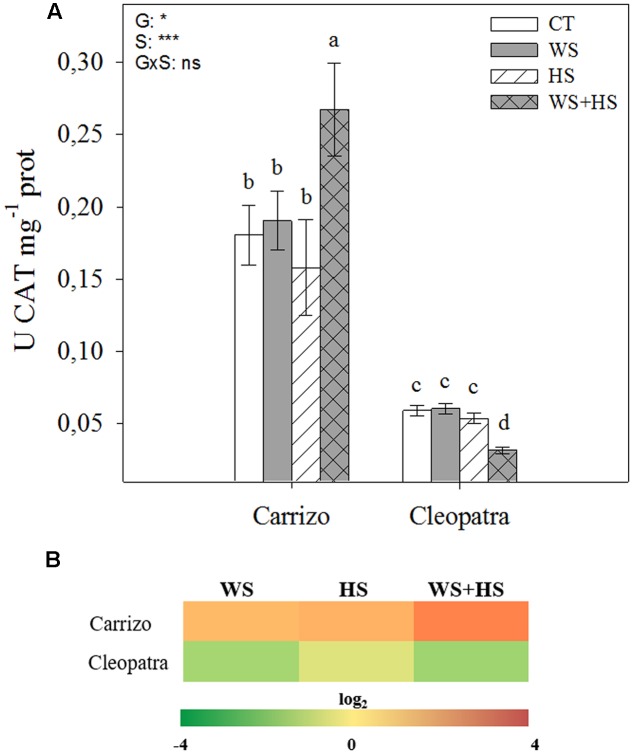
Effects of drought (WS), heat (HS), and a combination of drought and heat stress (WS+HS) on CAT activity **(A)** and transcript expression **(B)** in leaves of Carrizo and Cleopatra plants. Different letters denote statistical significance at *p* ≤ 0.05. G, genotypes; S, stress treatment; G×S, interaction genotype × stress treatment. ^∗^*P* < 0.05; ^∗∗^*P* < 0.01; ^∗∗∗^*P* < 0.001; ns, no statistical differences. Scale for gene expression is log_2_ of the mean values after normalization against control plants.

Similar to SOD, CAT activity was more than three-fold higher in Carrizo than in Cleopatra in all conditions studied. In response to individual drought and HS, CAT activity did not change with respect to control values in leaves of both citrus genotypes. Interestingly, under stress combination, CAT activity increased in Carrizo and decreased in Cleopatra compared to control levels (**Figure 5A**). Analysis of the relative accumulation of CAT transcripts in Carrizo revealed a remarkable up-regulation under individual and especially under combined stress conditions. Contrarily, CAT was down-regulated in Cleopatra leaves, particularly under WS and WS+HS (**Figure 5B**).

**FIGURE 6:**
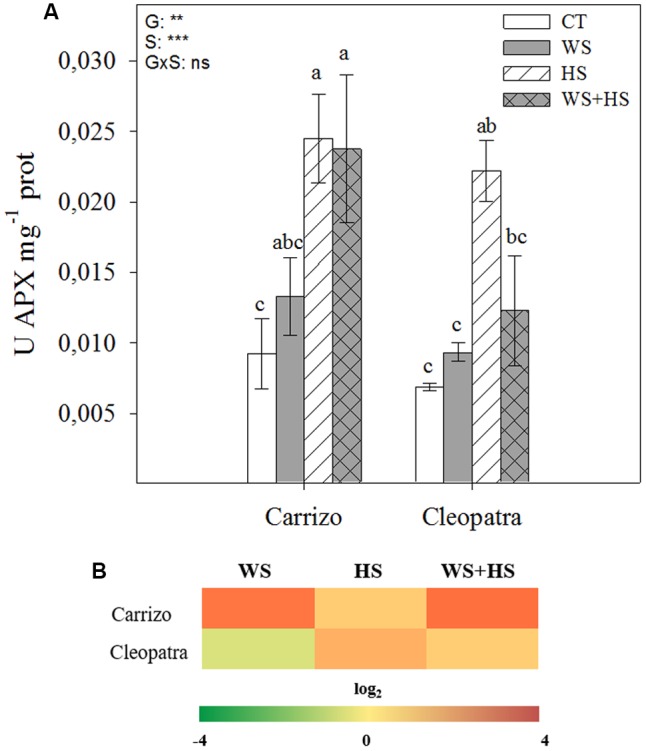
Effects of drought (WS), heat (HS), and a combination of drought and heat stress (WS+HS) on APX activity **(A)** and cytosolic APX transcript expression **(B)** in leaves of Carrizo and Cleopatra plants. Different letters denote statistical significance at *p* ≤ 0.05. G, genotypes; S, stress treatment; G×S, interaction genotype × stress treatment. ^∗^*P* < 0.05; ^∗∗^*P* < 0.01; ^∗∗∗^*P* < 0.001; ns, no statistical differences. Scale for gene expression is log_2_ of the mean values after normalization against control plants.

Ascorbate peroxidase activity significantly increased in response to HS and the combination of WS+HS with respect to control conditions in Carrizo leaves, whereas in Cleopatra a significant increment in APX activity was observed only in response to HS (**Figure 6A**). Moreover, the relative expression of cytosolic APX was up-regulated under HS and especially under WS and WS+HS in Carrizo, whereas only HS and WS+HS induced the accumulation of APX transcripts in Cleopatra (**Figure 6B**).

**FIGURE 7:**
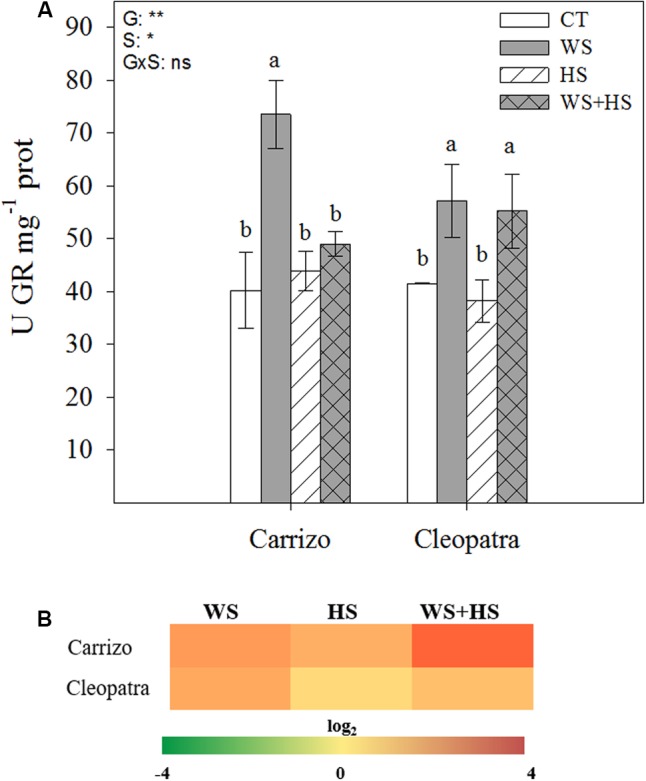
Effects of drought (WS), heat (HS), and a combination of drought and heat stress (WS+HS) on GR activity **(A)** and transcript expression **(B)** in leaves of Carrizo and Cleopatra plants. Different letters denote statistical significance at *p* ≤ 0.05. G, genotypes; S, stress treatment; G×S, interaction genotype × stress treatment. ^∗^*P* < 0.05; ^∗∗^*P* < 0.01; ^∗∗∗^*P* < 0.001; ns, no statistical differences. Scale for gene expression is log_2_ of the mean values after normalization against control plants.

In Carrizo plants, WS significantly increased the GR activity whereas neither HS nor WS+HS had effect on it. In contrast, in Cleopatra plants, WS and WS+HS increased GR activity and HS did not change this enzymatic activity respect to control levels (**Figure 7A**). Nevertheless, GR transcript number increased under all stress conditions studied in both genotypes, mainly in Carrizo leaves under stress combination (**Figure 7B**).

### AsA and GSH Pool

Under combined stress, tAsA and AsA levels increased in Carrizo and Cleopatra leaves with respect to control values (**Table 2**). Moreover, Cleopatra showed a higher tAsA content than Carrizo under combined stress conditions. However, DHA content only increased in Cleopatra leaves in response to stress combination. In addition, no significant alteration in leaf redox AsA/DHA ratio was observed within each citrus genotype (**Table 2**). Additionally, in response to stress combination, Carrizo and Cleopatra leaves accumulated significant higher levels of tGSH, GSH, and GSSG respect to control values (**Table 3**). Furthermore, HS induced an accumulation of tGSH, GSH, and GSSG compared to control conditions only in Carrizo leaves. GSH/GSSG ratio increased in Cleopatra leaves upon imposition of WS with respect to control values and higher values in this ratio were found in CT and WS conditions respect to Carrizo values (**Table 3**).

**Table 2:** Ascorbate (AsA), total ascorbate (tASA), and dehydroascorbate (DHA) content in Carrizo and Cleopatra leaves subjected to drought (WS), heat (HS), and their combination (WS+HS).

Genotype	tAsA (μmol g^-1^ FW)	AsA (μmol g^-1^ FW)	DHA (μmol g^-1^ FW)	AsA/DHA
**Carrizo**				
CT	4.68 ± 0.2 e	4.26 ± 0.09 b	0.42 ± 0.11 bc	11.58 ± 2.79 ab
WS	3.87 ± 0.31 e	3.58 ± 0.26 b	0.29 ± 0.05 c	13.11 ± 1.59 a
HS	7.24 ± 0.55 bc	6.0 ± 0.35 ab	1.24 ± 0.33 bc	5.04 ± 2.3 bc
WS+HS	9.35 ± 0.32 b	8.53 ± 0.33 a	0.81 ± 0.16 bc	10.51 ± 1.76 ab
**Cleopatra**				
CT	4.75 ± 0.74 de	3.91 ± 0.52 b	0.84 ± 0.39 bc	6.52 ± 1.99 abc
WS	7.02 ± 0.72 cd	4.82 ± 0.48 b	2.2 ± 0.39 b	2.40 ± 0.47 c
HS	6.12 ± 0.21 cde	4.13 ± 0.25 b	1.99 ± 0.18 bc	2.10 ± 0.23 c
WS+HS	13.84 ± 0.41 a	8.4 ± 1.18 a	5.44 ± 1.17 a	1.68 ± 0.35 c
	G: ^∗∗∗^	G: ^∗∗∗^	G: ^∗∗∗^	G: ns
	S: ^∗∗^	S: ns	S: ^∗∗∗^	S: ^∗∗∗^
	G×S: ns	G×S: ns	G×S: ^∗∗∗^	G×S: ns

*Data are presented as mean value of three different replicates ± SD. Different letters denote statistical significance at *p* ≤ 0.05. G, genotype; S, stress treatment; G×S, interaction genotype × stress treatment. ^∗^*P* < 0.05; ^∗∗^*P* < 0.01; ^∗∗∗^*P* < 0.001; ns, no statistical differences*.

**Table 3:** Total glutathione (tGSH), reduced glutathione (GSH), and oxidized glutathione (GSSG) content in Carrizo and Cleopatra leaves subjected to drought (WS), heat (HS), and their combination (WS+HS).

Genotype	tGSH (nmol g^-1^ FW)	GSH (nmol g^-1^ FW)	GSSG (nmol g^-1^ FW)	GSH/GSSG
**Carrizo**				
CT	96.5 ± 3.2 cd	81.2 ± 5.8 cd	15.4 ± 7.3 bc	4.5 ± 2.0 c
WS	83.4 ± 7.7 cd	75.7 ± 7.2 cd	7.7 ± 3.5 c	6.6 ± 0.4 c
HS	147.8 ± 5.5 a	118.3 ± 12.6 ab	29.4 ± 8.2 a	4.8 ± 1.5 c
WS+HS	153.5 ± 22.6 a	129.4 ± 22.7 a	24.1 ± 0.4 ab	5.4 ± 1.0 c
**Cleopatra**				
CT	77.8 ± 6.8 cd	73.5 ± 6.8 d	4.3 ± 0.1 c	17.0 ± 1.3 b
WS	75.5 ± 5.3 d	68.4 ± 8.2 d	7.1 ± 3.4 c	20.9 ± 1.8 a
HS	107.9 ± 5.5 bc	92.8 ± 4.8 bcd	15.1 ± 0.7 bc	6.1 ± 0.1 c
WS+HS	132.6 ± 4.2 ab	109.9 ± 3.7 abc	22.7 ± 1.3 ab	4.9 ± 0.3 c
	G: ^∗∗∗^	G: ^∗∗^	G: ^∗∗∗^	G: ^∗∗∗^
	S: ^∗∗^	S: ns	S: ^∗^	S: ^∗∗∗^
	G×S: ns	G×: ns	G×S: ns	G×S: ^∗∗∗^

*Data are presented as mean value of three different replicates ± SD. Different letters denote statistical significance at *p* ≤ 0.05. G, genotype; S, stress treatment; G×S, interaction genotype × stress treatment. ^∗^*P* < 0.05; ^∗∗^*P* < 0.01; ^∗∗∗^*P* < 0.001; ns, no statistical differences*.

## Discussion

Abiotic stresses including high temperatures, drought or different combinations of environmental challenges, induce metabolic imbalances that can cause an oxidative stress in plant cells. This effect results in the generation and accumulation of ROS, promoting oxidation of cellular components, hindering metabolic activities and affecting organelle integrity (Suzuki et al., 2012). In citrus plants, it has been proposed that environmental cues such as waterlogging, Cu toxicity, salinity or drought induce oxidative damage (Arbona et al., 2003, 2008; Wu et al., 2006; Hossain et al., 2009; Hippler et al., 2016), highlighting the importance of modulating the antioxidant system efficiently to cope with these abiotic stresses. In the present work, the antioxidant machinery of two citrus genotypes, Carrizo citrange and Cleopatra mandarin, with contrasting ability to tolerate the combination of drought and heat (Zandalinas et al., 2016b) was investigated to correlate differences in the modulation of the antioxidant system with tolerance to this stress combination. In this sense, Cleopatra constitutes a genotype more sensitive than Carrizo to drought combined with heat according to data presented in **Figure 1** and also reported in Zandalinas et al. (2016b). Therefore, the percentage of damaged leaves in response to heat or a combination of drought and heat was significantly higher in Cleopatra than in Carrizo (**Figure 1**), demonstrating the higher ability of Carrizo to deal with stresses that involve high temperatures. Moreover, a positive correlation between stress sensitivity and proline accumulation was observed. Hence, Cleopatra accumulated higher amounts of proline in leaves and roots in response to the combination of drought and heat compared to Carrizo. Additionally, both citrus genotypes accumulated higher levels of this metabolite in response to combined stress factors, a more damaging situation with respect to individual stresses. This result matches other works in which a higher stress pressure exerts a major proline accumulation (Claussen, 2005; Kaur and Asthir, 2015) due to its protective roles, including maintenance of redox balance and radical scavenging, maintenance of protein structure and contribution to reduce cell membrane damage (Shao et al., 2008; Szabados and Savouré, 2010).

In addition, oxidative damage (estimated by MDA accumulation) was also higher in leaves and roots of Cleopatra in response to combined stresses (**Figure 3**), suggesting that the extent of oxidative damage is directly linked to susceptibility of citrus plants to the combination of WS+HS. The increased SOD and CAT activities of Carrizo in both basal and stress conditions compared to Cleopatra (**Figures 4A**, **5A**) could be related to an active and efficient antioxidant response that might be involved in maintaining a lower MDA content (and oxidative stress, Gill and Tuteja, 2010) especially under the combination of drought and heat, and therefore helping citrus plants to cope with the combined stresses. On the contrary, whereas SOD activity of Cleopatra leaves increased in response to stress imposition, CAT activity did not change in response to individual stresses and even decreased below control levels under WS+HS conditions. In addition to CAT, APX removes H_2_O_2_ and this reaction has been previously reported to be a crucial process for the tolerance of plants to combined drought and heat (Koussevitzky et al., 2008). In our work, APX activity was significantly induced by HS and WS+HS in Carrizo leaves (**Figure 6A**), suggesting an efficient H_2_O_2_ scavenging ability under these stress conditions. However, in Cleopatra, only HS significantly induced an increased APX activity (**Figure 6A**). However, under high temperatures, this enzyme activity could be insufficient to scavenge the excess of H_2_O_2_ when CAT activity is not activated (**Figure 5A**), rendering an increased oxidative damage. Additionally, APX dismutase H_2_O_2_ using AsA as the electron donor (Foyer and Noctor, 2011). Both citrus genotypes showed increases in leaf AsA and tAsA contents in response to WS+HS, suggesting that the accumulation of AsA could be related with a stronger stress pressure. In addition, Cleopatra showed higher tAsA and DHA levels as well as a lower AsA/DHA ratio with respect to Carrizo during this stress combination (**Table 2**), which are according to the lower APX activity observed during WS+HS in this citrus genotype (**Figure 6A**).

Accurate modulation of the GSH cycle is involved in maintaining a favorable GSH/GSSG ratio required for cellular redox regulation. In this way, GR activity could effectively recycle GSH at the expense of NADPH (Foyer and Noctor, 2011). The pattern observed for GR activity in **Figure 7** indicates that Carrizo plants, despite increasing tGSH, GSH, and GSSG levels in response to HS and WS+HS (**Table 3**), preserved the GR activity as well as the GSH/GSSG ratio around control values, probably as a result of a lower incidence of the oxidative damage. In contrast, in Cleopatra leaves, the reduction in GSH/GSSG ratio with respect to control values, especially under WS+HS (**Table 3**), suggests an impairment of GSH recycling. This result points to a better ROS non-enzymatic detoxification system and to an efficient GSH recycling in Carrizo plants compared to Cleopatra, with no apparent NADPH limitation. In this sense, it has been previously reported that maintenance of a more GSSG status could be a consequence of an enhanced ROS accumulation (Foyer and Noctor, 2011). Our results are in accordance with this statement since MDA specially accumulated in Cleopatra in response to WS+HS (**Figure 3**). Furthermore, the activation of GR activity observed in Cleopatra under this stress combination (**Figure 7A**) might be insufficient to keep a proper GSH/GSSH ratio, leading to a lower ability for ROS detoxification (Arbona et al., 2008) and, as a result, to a higher sensitivity of this citrus genotype to WS+HS. These results also demonstrated the previous hypothesis, suggesting that a deficient antioxidant system in Cleopatra plants under the combination of drought and heat would lead to an enhanced activation of secondary metabolites with antioxidant properties including flavonols, flavones and limonoids to supplement the antioxidant deficiency and mitigate the damaging effects of stress (Zandalinas et al., 2017b). However, all these metabolic strategies, including proline accumulation (**Figure 2**), do not seem to be effective as Cleopatra mandarin suffered important damage under WS+HS conditions.

According to our data, the combination of drought and heat negatively impacted both citrus genotypes (**Figure 1**) but the effective activation of the antioxidant machinery was associated to the ability to tolerate this stress combination. As a result, in Cleopatra plants in response to WS+HS, the increment of SOD activity (**Figure 4A**) along with the decline in CAT activity (**Figure 5A**) and the lack of APX activity increase (**Figure 6A**), compared to control values could be partially responsible of its increased oxidative damage and sensitivity to the combination of drought and high temperatures. In contrast, the ability of Carrizo plants to efficiently activate antioxidant enzymes involved in ROS detoxification along with preserving a favorable GSH/GSSG ratio would be partially related to genotype tolerance to combined stresses. This work provides physiological basis for directing future genetic programs to improve the antioxidant system of Cleopatra mandarin, a genotype that has been very useful as a rootstock for plants cultivated under conditions of water scarcity. However, its future use can be seriously compromised in a scenario of climatic change due to the high sensibility to combined conditions of heat and drought.

## Author Contributions

SZ and DB performed the research. AG-C and VA supervised the project and provided funding. SZ, DB, and AG wrote the manuscript and prepared figures. SZ, DB, VA, and AG-C revised the final version. All authors have read and approved the final version of the manuscript.

## Conflict of Interest Statement

The authors declare that the research was conducted in the absence of any commercial or financial relationships that could be construed as a potential conflict of interest.

## Abbreviations

ABA: abscisic acid
APX: ascorbate peroxidase
AsA: ascorbate
CAT: catalase
CT: control
GR: glutathione reductase
GSH: glutathione
GSSG: oxidized glutathione
HS: heat stress
M_d,_: dry mass
MDA: malondialdehyde
M_f,_: fresh mass
M_t,_: turgid mass
PP2Cs: protein phosphatases 2C
REST: Relative Expression Software Tool
ROS: reactive oxygen species
RWC: relative water content
SOD: superoxide dismutase
WS: water stress
WS+HS: water stress and heat stress combination.
