# Supplementary material for: Modulation of Antioxidant Defense System Is Associated with Combined Drought and Heat Stress Tolerance in Citrus
Source: Front Plant Sci. 2017 Jun 7;8:953. doi: 10.3389/fpls.2017.00953 (PMC5461256; doi:10.3389/fpls.2017.00953)
Supplement: Supplementary file 1 [file Table_1.DOCX]

| **Citrus gene** | **Locus** | **Forward /**  **Reverse** | **Sequence (5’🡪3’)** | **Amplicon size (bp)** |
| --- | --- | --- | --- | --- |
| **SOD-CuZn** | orange1.1g031837m | F | CTTGGTGGAACTGAGGGTGT | 173 |
|  |  | R | GGGTTAAAGTGGGGTCCAGT |  |
| **SOD-Fe** | orange1.1g026199m | F | CAGCTTCATCTGCTCCAACA | 148 |
|  |  | R | GGTGGAGGCTTCAAATCAAA |  |
| **CAT** | orange1.1g042356m | F | GTAACCAAGACCTGGCCTGA | 134 |
|  |  | R | ATGCCAGGAACCACAATAGC |  |
| **APX** | orange1.1g024615m | F  R | CCATTCGGAACCATGAGGCT CTCAACGCCAACAACACCAG | 153 |
| **GR** | orange1.1g042564m | F  R | CTTGGAGCATCAATGTGTGG AGCAACACGTCTCGTCACAG | 165 |
| **ACT** | orange1.1g037845m | F | CCCTTCCTCATGCCATTCTTC | 105 |
|  |  | R | CGGCTGTGGTGGTAAACATG |  |
| **TUB** | orange1.1g013335m | F | GGGGCAAAATGAGCACTAAA | 187 |
|  |  | R | CGCCTGAACATCTCCTGAAT |  |

**Table S1**. Designed primers for gene expression analyses by quantitative RT-PCR.
